# On Some of the Furniture of a Sick Room

**Published:** 1803-08-01

**Authors:** 


### *On some of the* FURNITURE *of a* SICK ROOM.

THERE is no part of the furniture of a sick chamber that calls more importunately for reform than close stools. The

The great fault in the structure of them is their being contrived for more purposes than one; in the chambers of the wealthy, in particular, we see them become a piece of furniture, as Dean Swift says, "with rings and hinges counterfeit;" and being a piece of elegant furniture it must remain in the room; the pan and contents only are removed, and this very frequently not until some hours after being used, during which time the wood is imbibing the putrid effluvia.

I shall now beg leave to speak more particularly of the structure of those close-stools in general use with the middling and lower class of people, and to which this paper more especially applies. They are made square, and when you are called upon to visit a patient as the lid is a flat surface, you will observe it take the place of a chair by the bed-side, and being so flat it generally answers the purpose of a table; also this nauseous putrid apparatus is placed directly under the nose of the patient, where it continues without the least exposure to air or fumigation during the whole illness. I have known the stench complained of by the patient. The nurse answers, "I am sure I have emptied it;" and, with few exceptions, neither the nurse nor the patient have the least idea of the close-stool itself being the cause. The sick person then is persuaded to be reconciled; he conceives he must be mistaken in *imagining* a stench, although he is nearly poisoned with it. If perchance however the patient is positive that the stench is in the close stool, he becomes tired of complaint, as the nurse remains obstinate in her assertion, and perhaps will once or twice at most growlingly remove it; the sick patient is then hurt in two ways, the mind is irritated, and he breathes an unfit air. We know there are diseases where the sense of smelling becomes more acute, the phthisis pulmonalis for example; and the attentive practitioner will be cautious in visiting his patient a second time in boots after they have once been complained of.

How frequently with the poorer class will one close-stool go through a whole parish; I have known the precious chest brought from a neighbour who had just expired upon it, into the chamber of another who was immediately fixed there.

I shall conclude with the alteration I propose. In the upper part the lid should be convex; this will prevent its being applied to more uses than one, no person can make a chair of it to sit upon; it will not hold barley-water, or other drink, it will consequently be removed from the bed-side, side, which is one point at least gained, if it never gets fumigated, and the lid should not be attached to the close-stool by hinges, but should lift off, to allow the patient to lean his back against any thing.

Much more might be said on the subject, but I am addressing myself to those who will readily comprehend my meaning; and nauseous as the subject at the first blush may appear, I trust it will be found to be of material advantage.

